# Inhibition of androgen-responsive LNCaP prostate cancer cell tumor xenograft growth by dietary phenethyl isothiocyanate correlates with decreased angiogenesis and inhibition of cell attachment

**DOI:** 10.3892/ijo.2012.1335

**Published:** 2012-01-17

**Authors:** TAMARO S. HUDSON, SUSAN N. PERKINS, STEPHEN D. HURSTING, HEATHER A. YOUNG, YOUNG S. KIM, TIEN-CHUNG WANG, THOMAS T.Y. WANG

**Affiliations:** 1 Laboratory of Cellular Regulation and Carcinogenesis, National Cancer Institute, National Institutes of Health, Bethesda, MD 20892; 2 Department of Nutritional Sciences, University of Texas at Austin, Austin, TX 78712; 3 Department of Carcinogenesis, University of Texas-M.D. Anderson Cancer Center, Smithville, TX 78957; 4 Division of Epidemiology and Biostatistics, The George Washington University School of Public Health and Health Services, Washington, DC 20037; 5 Nutritional Sciences Research Group, Division of Cancer Prevention, National Cancer Institute, National Institutes of Health, Bethesda, MD 20892; 6 Department of Nutrition and Food Science, University of Maryland, College Park, MD 20742; 7 Diet, Genomics and Immunology Laboratory, Beltsville Human Nutrition Research Center, US Department of Agriculture, Beltsville, MD 20705, USA

**Keywords:** angiogenesis, phenethyl isothiocyanate, prevention, prostate cancer, xenograft

## Abstract

Phenethyl isothiocyanate (PEITC) is a candidate anticancer compound found in certain cruciferous vegetables. In our tumor cell xenograft model, dietary administration of PEITC (100–150 mg/kg body weight/d) inhibited androgen-responsive LNCaP human prostate cancer cell tumor growth. We found that dietary treatment with PEITC significantly inhibited tumor platelet/endothelial cell adhesion molecule (PECAM-1/CD31) expression, a marker of angiogenesis. By contrast, we did not find the inhibitory effects of PEITC on tumor growth to be associated with alteration of specific markers for apoptosis, cell proliferation or androgen receptor-mediated pathways. Consistent with *in vivo* results, PEITC exerted little effects on cell proliferation, cell cycle and androgen-dependent pathways. Interestingly, PEITC significantly attenuated LNCaP cell plating efficiency that correlated with inhibition of integrin family proteins integrin β1, α2 and α6 mRNA expression. Thus, PEITC may be a dietary factor that inhibits androgen-responsive prostate tumor growth indirectly by selectively targeting factors involved in the tumor microenvironment.

## Introduction

Prostate cancer is one of the most insidious diseases in men in the Western hemisphere. Moreover, the incidence and mortality among men in industrialized Western countries is higher than in Asian men (1). The critical factor may be lifestyle and diet, specifically low consumption of the fruits and vegetables that contain many phytochemical agents (2–7). In addition to the high incidence and mortality, there remains a lack of satisfactory treatment options for metastatic prostate cancer. Therefore, prostate cancer is an ideal candidate disease for chemoprevention. Accordingly, there is intense effort to identify potential naturally occurring and synthetic agents with anticancer and anti-angiogenic activity (8,9).

Isothiocyanates (ITC), which are abundant in foods derived from cruciferous vegetables, are one of the most studied classes of chemopreventive agents (10). ITCs are released from glucosinolate precursors after hydrolysis by the enzyme myrosinase, which is activated during injury of the plant (11). There are over one hundred naturally occurring ITCs, and, of these, phenethyl isothiocyanate (PEITC) has received the most attention because of its ability to inhibit CYP enzymes, decrease cell survival, induce apoptosis *in vitro*, and attenuate tumor burden. For instance, in human HeLa cells, PEITC, as well as other isothiocyanates, has been shown to induce apoptosis through a capase-3-dependent mechanism (12). Additionally, cysteine protease (i.e., caspase-3 and -8) activity increases in human leukemia cells during PEITC-induced apoptosis (13). Most importantly, it has been reported that PEITC inhibits metastatic prostate cancer cell growth. In the androgen-independent PC-3 metastatic prostate cancer cell line, PEITC inhibits cell survival and increases apoptosis, effects that are mediated by activation of extracellular signal-regulated kinases (ERK1/2) (14). In addition, PEITC significantly inhibits phosphorylation of both IKKα and IKKβ in PC-3 cells (15). Moreover, PEITC treatment has been shown to inhibit the growth and migration of endothelial cells and the formation of new blood vessels (16). However, the effects of PEITC on the early prostate carcinogenesis process, as represented by androgen-responsive tumors, are not known. From a preventive perspective it is important to delineate the effects on this earlier stage.

In this study, we tested the effects of PEITC on cell proliferation, apoptosis, angiogenesis, and androgen-dependent signaling pathways in androgen-responsive LNCaP human prostate cancer cell xenografts in order to elucidate mechanisms that may help explain the prostate cancer preventive effects of PEITC. We also utilize cell culture model to examine potential mechanisms of action of PEITC. We found that PEITC may inhibit androgen-dependent prostate tumor growth by targeting angiogenesis and cell attachment.

## Materials and methods

### Chemicals and diets

PEITC (indicated purity 99%), dimethylsulfoxide (DMSO), and propidium iodide were purchased from Sigma-Aldrich Chemical Co. (Milwaukee, WI). Powdered AIN-93M diets with or without PEITC [3 μmol/g diet (17)] were prepared by Research Diets (New Brunswick, NJ) and stored at −20°C until weekly feedings.

### Cells and cell culture

LNCaP human prostate cancer cells were obtained from the American Type Culture Collection (Manassas, VA) and grown in RPMI-1640 supplemented with 2 mM L-glutamine, 10% fetal bovine serum (FBS), 100 U/ml penicillin, and 100 μg/ml streptomycin (Invitrogen, Carlsbad, CA) at 37°C in a 5% CO_2_ atmosphere.

### In vivo xenograft bioassay

All experimental protocols were in accordance with the National Institutes of Health guidelines and were approved by the USDA Animal Research Advisory Committee. Male athymic nude mice (BALB/c nu/nu, 20–22 g, 5–6 weeks old; Charles River, Frederick, MD) were individually housed in filter-top cages at the USDA BHNRC animal facility and consumed food and fresh tap water *ad libitum*. Food consumption and body weights were recorded weekly. After an acclimation period of 1 week, during which mice were fed control AIN-93M diet, the mice were randomized into 2 experimental groups, with 19 animals in the control group and 22 animals in the PEITC treatment group. Two weeks later, LNCaP human prostate cancer cell xenografts were established in the mice by injection s.c. in the flank with LNCaP cells (2×10^6^ cells) in 50 μl of phosphate-buffered saline (PBS) plus 50 μl Matrigel (BD Biosciences, Mansfield, MA). This produced palpable tumors within 4–5 days. Cancer preventive efficacy of the treatments was assessed once a week by measuring tumor volume (cm^3^) = 0.523 × [length (cm) × width^2^ (cm^2^)] (18). Mice remained on their respective diets for 7 weeks after cell injection, until tumors reached 2–3 cm^3^ in volume, and the animals were then sacrificed. A portion of tumor tissue was fixed in 10% neutral buffered formalin for *in situ* immunohistochemical analysis and quantitation of proliferation, apoptosis, and angiogenesis as previously described (19). A portion of tumor tissue was quickly frozen in liquid nitrogen and stored at −70°C for mRNA and protein analysis as described below.

### Cell proliferation and cell attachment assay

LNCaP cells (1–5×10^5^ cells/well) were plated in 24-well plates (Corning Life Sciences, Corning, NY) and 24 h later were treated with 0 (vehicle, DMSO), 0.1, 1, or 5 μM PEITC for 0–75 h, with the medium replaced every 24 h. Cell growth was analyzed using the sulforhodamine B (SRB) assay as described previously (22). Results are expressed as mean absorbance plus or minus standard error of the mean (mean ± SE). For cell attachment assays, LNCaP cell were harvested by trypsinization and re-suspend in media containing 0, 0.5, 1, 2.5 or 5 μM of PEITC at 1×10^5^ cells/ml. Cells were immediately plated in 24-well-plates (1×10^5^ cell/well). After overnight incubation, media were removed from the wells and the wells were wash once with PBS, fixed with 10% trichloroacetic acid. Cells attached to culture plate surface were determined by SRB method (22).

### FACS analysis of cultured cells

LNCaP cells were plated in duplicate at a density of 1×10^5^ cells/well in 6-well plates (Corning), which were incubated for 24 h and subsequently synchronized by culturing without serum for 24 h; results are from two independent experiments. Based on the results from the cell growth assays, the cells were then treated with PEITC at 5 or 10 μM for 24, 48, and 72 h. The cells were fixed and stained with propidium iodide for analysis by flow cytometry (FACSCalibur, Mansfield, MA), and the results were evaluated using FlowJo software (BD Biosciences, San Jose, CA).

### RNA isolation and reverse transcription (RT)-PCR in cultured cells and tumors

LNCaP prostate cancer cells were plated in 6-well plates (1×10^6^ cells/well) and 24 h after plating were switched to Media B [RPMI-1640 medium without phenol red (Invitrogen), 2 mM L-glutamine, 100 U/ml penicillin, 100 μg/ml streptomycin, and 10% charcoal dextran-treated FBS (Hyclone, Logan, UT)] to minimize the effect of serum hormones. Twenty-four hours later, the medium was replaced with fresh medium containing 1 nM dihydrotestosterone (DHT) with or without 0–5 μM PEITC. The medium was changed daily to fresh medium with test compounds, and after 48 h cells were harvested for total RNA isolation using the TRIzol method (Invitrogen) as described previously (22). For determination of mRNA expression in tumor samples, tumor total RNA was isolated using the TRIzol (Invitrogen) method following the manufacturer's protocol. Gene expression was quantified by the TaqMan real-time RT-PCR method as described previously (22). TaqMan gene expression assay primers and probes for human prostate specific antigen (PSA) and glyceraldehydes-3-phosphate dehydrogenase (G3PDH), proliferating cell nuclear antigen (PCNA), Ki-67, integrin β1 (ITGB1), integrin α2 (ITGA2), integrin α5 (ITGA5), integrin α6 (ITGA6), mouse and human vascular endothelial growth factor (VEGF) A, mouse PECAM-1 and mouse and human TATA binding protein were purchased from Applied Biosystems (Foster City, CA). G3PDH was used as a housekeeping gene for calculation of relative expression levels *in vitro* and mouse or human TATA binding protein was used for tumor samples.

### Western blot analysis of tumor samples

Tumor tissue from 4 mice in each dietary treatment group was lysed with T-PER Tissue Protein Extraction Reagent (Pierce, Rockford, IL) supplemented with protease inhibitors (Complete Mini Protease Inhibitor Cocktail Tablets (Roche Applied Science, Indianapolis, IN), 50 mmol/l sodium fluoride, and 1 mmol/l sodium orthovanadate) and homogenized. Total cell lysate proteins (30 μg) were separated using 4–20% or 8–16% pre-cast denaturing polyacrylamide Tris-glycine gels (Invitrogen) and transferred by iBlot machine (Invitrogen) onto PVDF membranes (Invitrogen). The membrane was probed or with antibodies (all from Santa Cruz Biotechnology, Santa Cruz, CA) against human caspase-3 (clone H-277), human VEGF (clone 147), diluted 1:200 overnight at 4°C, and developed using the WesternBreeze Chemiluminescent Immunodetection Kit (Invitrogen) according to the manufacturer's instructions. Two blots were run for each antibody. Blots were stripped and re-probed with anti-β-actin (Chemicon International, Inc., Temecula, CA) as a loading control. Immunoreactive bands were visualized by X-ray film (Kodak X-Omat MR-1) and digitized using a flatbed scanner (Hewlett-Packard model 4850, Hewlett-Packard, Palo Alto, CA) according to the manufacturer's protocol. The intensity of the band was determined using ImageJ software (http://rsb.info.nih.gov/ij/).

### Statistical analysis

In our *in vivo* experiments StatView (SAS Institute, Cary, NC) software was used for statistical analysis. Repeated measures multivariate analysis of variance with Wilks' λ as the test statistic was used to compare differences between control and PEITC groups in time-to-appearance of palpable tumor and rate of tumor growth between control and PEITC groups over time. The 19–22 animals used per group gave >97% power to detect effects and provided ≥85% power to detect differences as small as 35%. For our *in vitro* experiments, the GraphPad PRISM 4 (GraphPad Software Inc. San Diego, CA) was used. The unpaired Student's t-test was used to compare experiments with two groups. ANOVA followed by Bonferroni's multiple comparison *post hoc* test was used to examine differences in PSA mRNA levels between DHT-treated and DHT-non-treated groups.

## Results

### PEITC inhibits tumor growth in an LNCaP prostate cancer cell xenograft model

**Figure 1:**
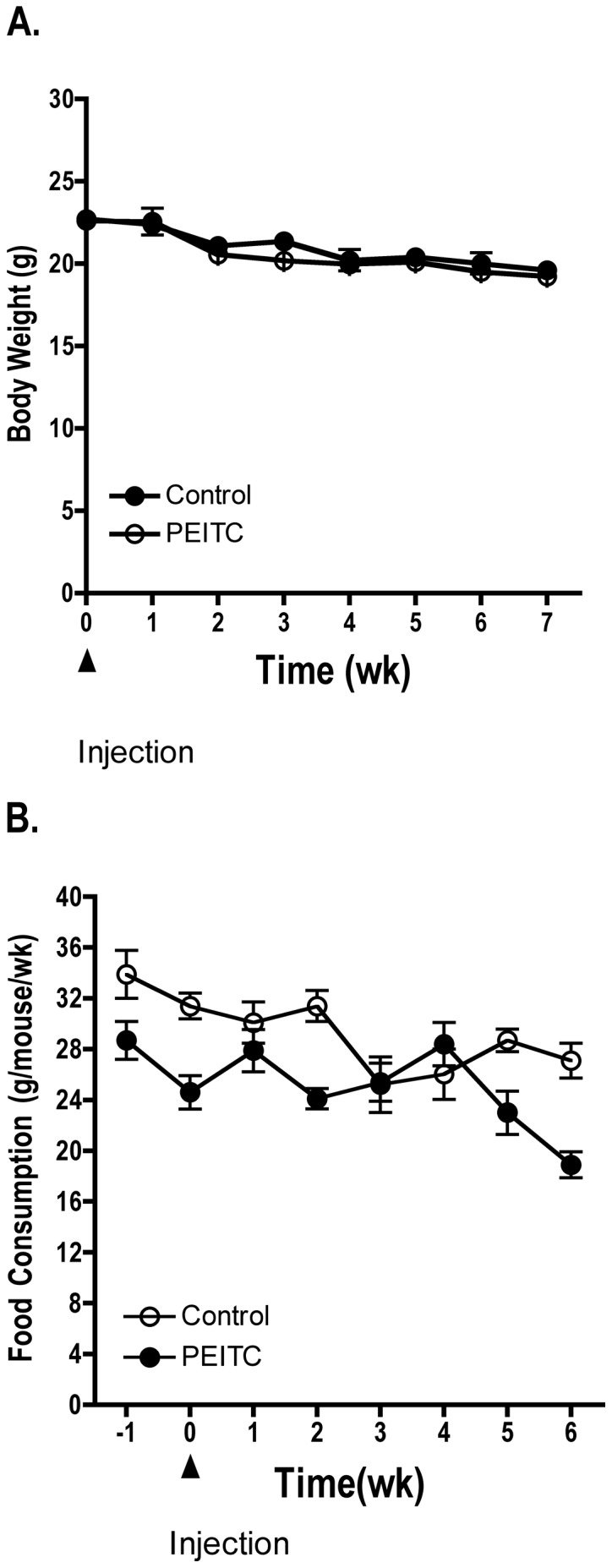
Dietary administration of PEITC did not affect body weight or food consumption of athymic nude mice. Athymic nude mice were fed either control or PEITC diet (3 μmol/g diet) for 2 weeks before injection of LNCaP human prostate cancer cells at week 0. Mice were euthanized after 7 additional weeks on their respective diets. (A) Mean weekly body weights of control and PEITC-treated mice. (B) Mean weekly food consumption of control and PEITC-treated mice. Values are means ± SE. Control, n=19; PEITC, n=22.

Dietary administration of PEITC (3 μmol/g diet) did not significantly affect body weight or food consumption of treated mice compared to control mice (Fig. 1). However, during the final weeks of the experiment mice in both the control and treated groups experienced a slight decrease in body weight and food consumption as the LNCaP prostate cancer cell xenografts grew in size in both the control and treated groups. Each mouse in the treatment group consumed ~100–150 mg/kg body weight/day of PEITC. Four mice from each group died prior to study termination. Overall, administration of PEITC to athymic nude mice did not present any observable toxicity as determined by pathological analysis of kidney and liver from the mice (data not shown).

**Figure 2:**
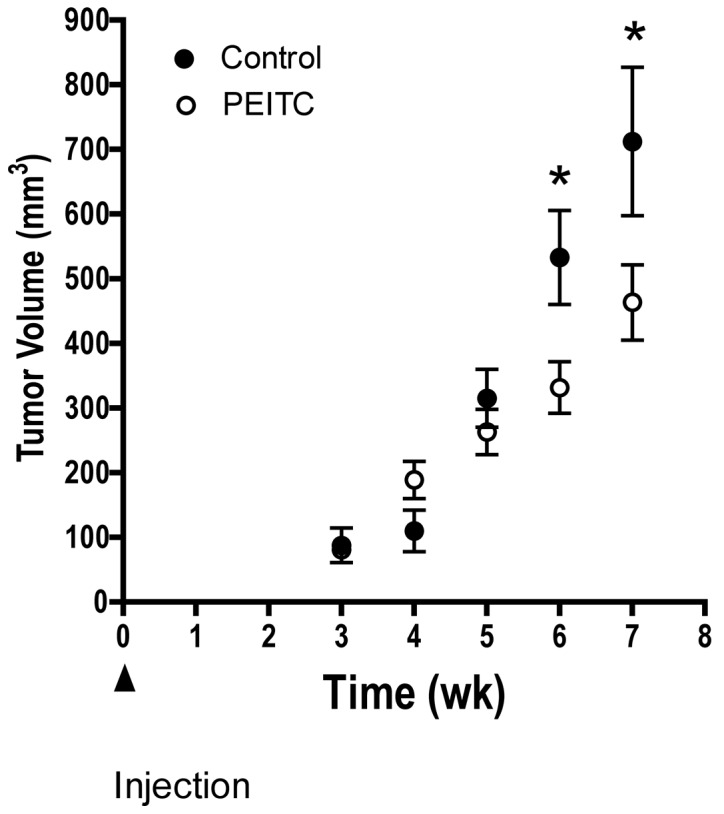
Dietary administration of PEITC decreased growth of LNCaP prostate cancer cell xenografts in athymic nude mice. LNCaP human protate cancer cell xenografts were established in athymic nude mice, and tumor volume was measured weekly and calculated as described in Materials and methods. Values are means ± SE. Control, n=19; PEITC, n=22. ^*^Significant difference between control and PEITC treatment at p<0.05.

Fig. 2 illustrates the temporal effects of dietary PEITC on tumor growth in mice fed either control or PEITC diet. We observed a significant delay in tumor growth in mice consuming PEITC compared to control diet. The difference was observed at 6 and 7 weeks after injection of LNCaP prostate tumor cells.

### PEITC does not affect apoptosis, cellular proliferation, or androgen-dependent pathways in vivo

**Figure 3:**
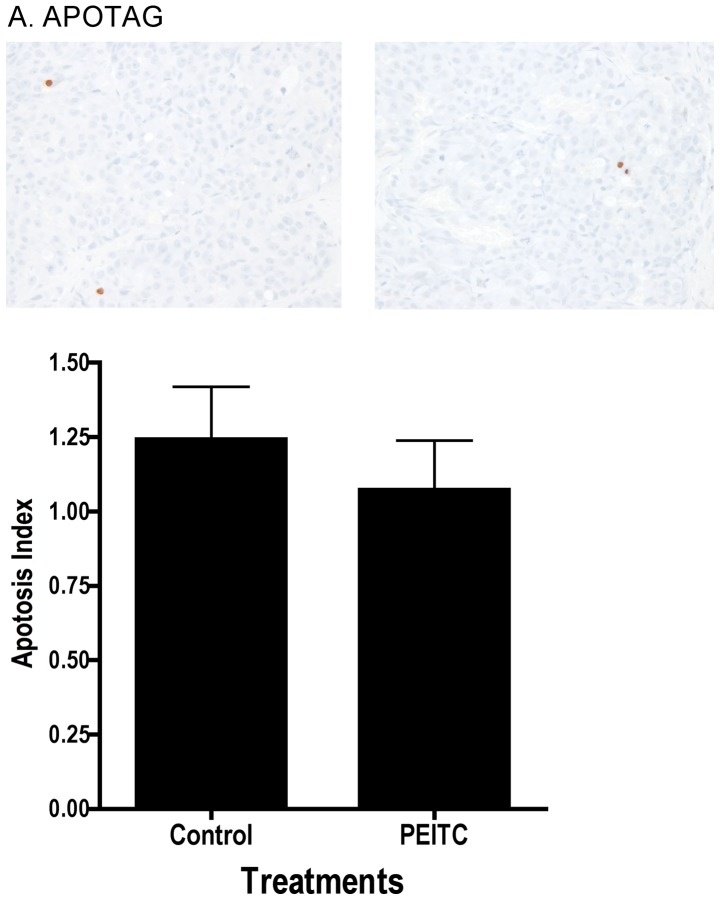

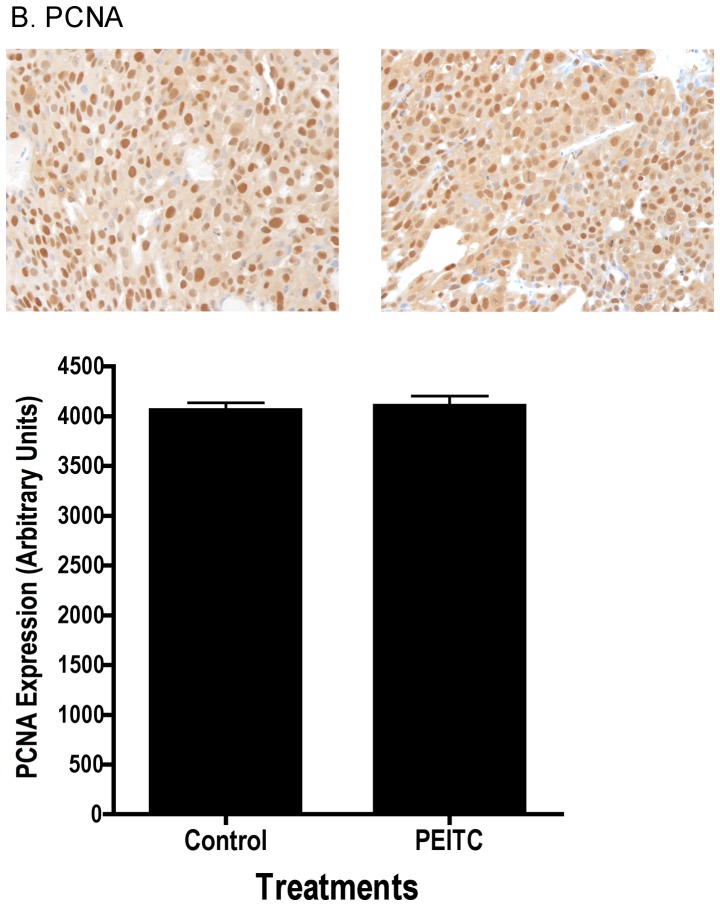
Dietary administration of PEITC does not induce apoptosis or decrease cellular proliferation in LNCaP prostate cancer cell xenografts in athymic nude mice. Immunohistochemical analysis of markers for apoptosis and proliferation was performed in paraffin-embedded tumor samples from control and PEITC-treated mice. All images were calibrated to a master (single image) color profile to maximize accuracy. (A) ApoTag, apoptosis index = average apoptosis-positive cells/field ± SE. (B) PCNA, proliferation index = average PCNA-positive cells/mm^2^ ± SE. Representative images of immunohistochmical staining are illustrated on top of bar graphs. Control, n=19; PEITC, n=22.

**Figure 4:**
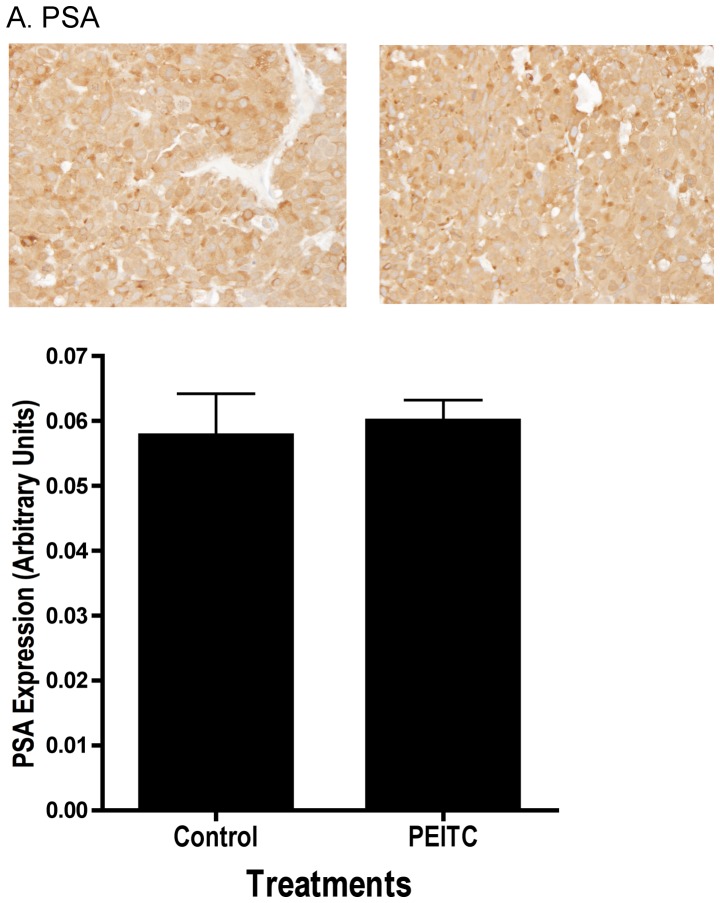

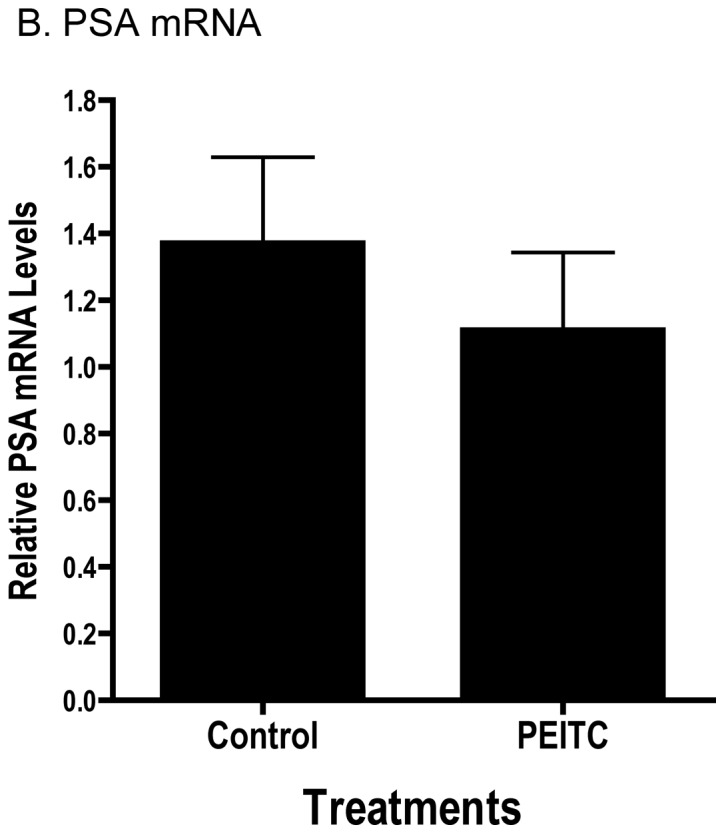
PEITC had no effect on PSA protein and mRNA expression in LNCaP xenografts *in vivo*. (A) PSA protein expression in LNCaP tumor xenograft. Immunohistochemical analysis of marker for PSA in paraffin-embedded LNCaP prostate cell tumor samples from xenografts in control and PEITC-treated mice were performed. No post-capture modification was done to the images in order to preserve the color intensity of each sample, and a 3-tier intensity scale was arbitrarily constructed to sort the varying degree of positive cells into groups. Relative PSA levels = average PSA positive cells/mm^2^ ± SE. A representative image of immunohistochemical staining is illustrated on top of bar graph. Control, n =19; PEITC, n=22. (B) PSA mRNA expression in LNCaP tumor xenograft. Real-time PCR analysis of PSA mRNA expression in tumor samples was performed as described in Materials and methods. Results are expressed as means ± SE (n=4).

**Figure 5:**
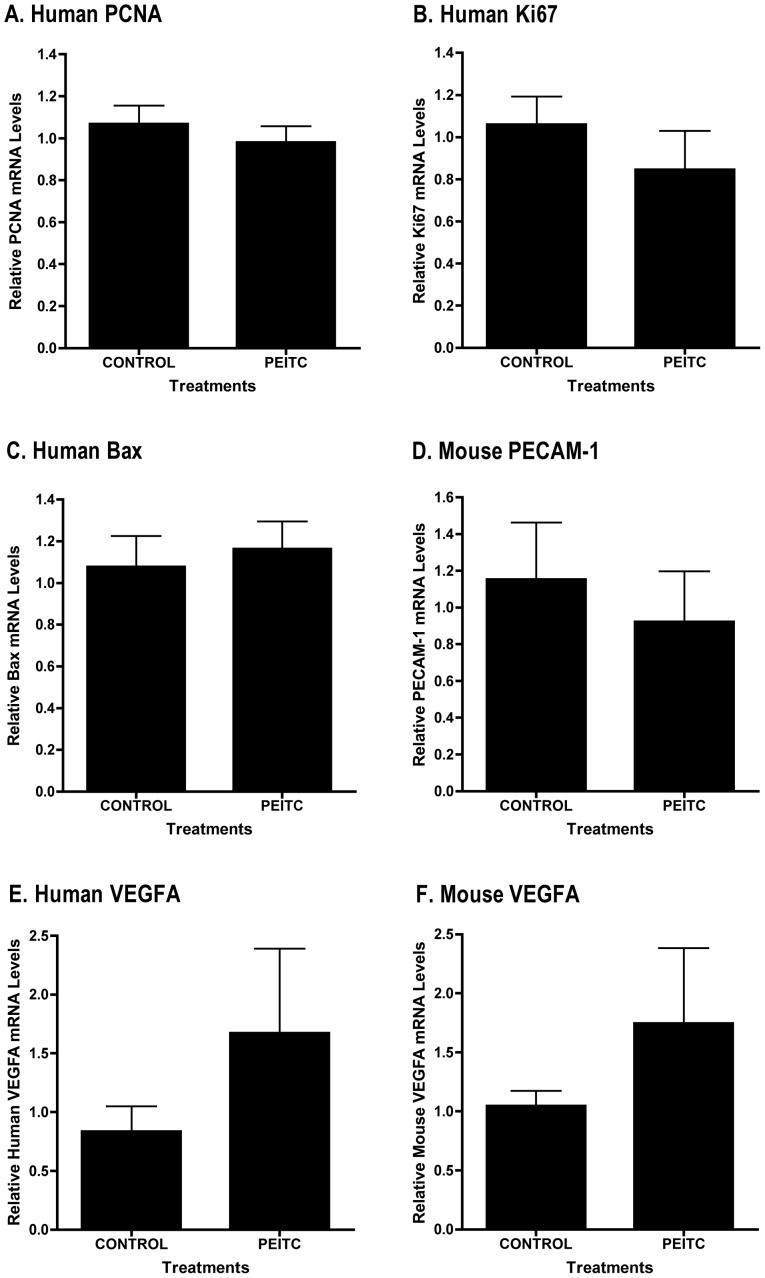
Real-time PCR analysis of mRNA expression of selective markers in control and PEITC-treated tumors. Real-time PCR analysis of gene expression was performed as described in Materials and methods. Results are expressed as mean ± SE (n=4). (A) Human PCNA. (B) Human Ki-67. (C) Human Bax. (D) Mouse PECAM-1. (E) Human VEGFA. (F) Mouse VEGFA.

**Figure 6:**
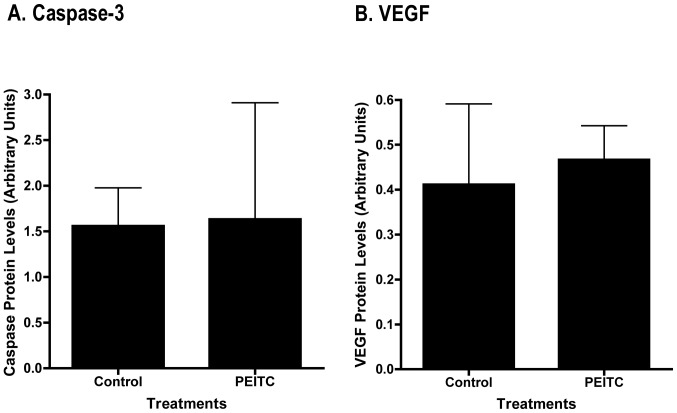
Western blot analysis of caspase-3 and VEGF in control and PEITC-treated tumors. Western blot analysis of caspase-3 and human VEGF were performed and quantitated as described in Materials and methods. Results were normalized to β-actin levels and expressed as means ± SE (n=4). (A) Caspase-3. (B) VEGF.

We evaluated whether the reduced rate of tumor growth with PEITC treatment was due to induction of apoptosis or a decrease in proliferation in the tumor. As illustrated in Fig. 3A, dietary treatment of mice with PEITC did not lead to changes in apoptosis index in the tumor as determined by TUNEL assay, nor did it affect the intensity of cellular proliferation marker PCNA (Fig. 3B). In addition to cell proliferation and apoptosis, we examined the effects of PEITC on androgen-dependent pathways, as androgen is a known risk factor for prostate cancer and is associated with increased proliferation and decreased apoptosis. We found the androgen responsive gene PSA protein levels in the tumor were not affected by PEITC as determined by immunohistochemistry (Fig. 4A). Additional analyses using real-time PCR and Western blot analyses were performed on tumor samples to confirm the results of immunohistochemical analysis. We also observed no differences in mRNA levels of PSA (Fig 4B), the proliferation markers PCNA (Fig. 5A) or Ki-67 (Fig. 5B) between control (n=4) and PEITC-treated (n=4) tumor samples. We found no change in pro-apoptosis protein Bax mRNA levels (Fig. 5C). We detected pro, but not processed (cleaved) caspase-3 in the tumor samples by Western blot analysis, and densitometric quantitation of the bands revealed no significant differences between control and PEITC-treated mice (Fig. 6A).

### PEITC attenuated microvessel density in tumor xenografts

**Figure 7:**
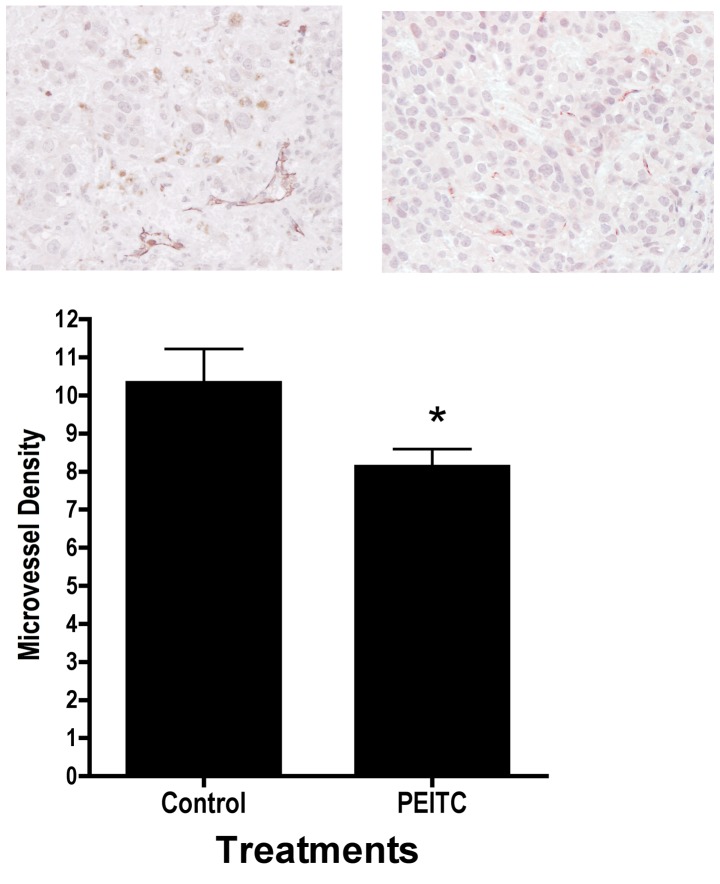
PEITC decreases microvessel density in LNCaP prostate cancer cell xenografts in athymic nude mice. Immunohistochemical analysis for the angiogenesis marker PECAM-1/CD31 was performed on paraffin-embedded tumor samples from control and PEITC-treated mice. All images were calibrated to a master (single image) color profile to maximize accuracy. Vessels were analyzed through contrast enhancement algorithms, and non-specific/background signal was filtered out via morphological algorithms. ^*^Significant difference between control and PEITC treatment at p<0.05. Angiogenesis index = average vessel counts/field ± SE. A representative image of immunohistochemical staining is illustrated on top of bar graph. Control, n=19; PEITC, n=22.

In addition to cellular proliferation and apoptosis, the tumor microenvironment may also affect prostate tumor growth. Therefore, we examined the effects of PEITC on microvessel density. As illustrated in Fig. 7, treatment with PEITC resulted in significantly lower microvessel density, as indicated by PECAM-1/CD31 staining intensity in the tumor. We were not able to detect the mouse PECAM-1 using Western blot analysis. However, real-time PCR analysis showed no difference in mouse PECAM-1 mRNA expression between control (n=4) and PEITC-treated (n=4) tumor samples (Fig. 5D). We also found no difference in mouse or human VEGF mRNA expression between control (n=4) and PEITC-treated (n=4) tumor samples (Fig. 5E and F) or in mouse or human VEGF expression using immunohistochemical detection (data not shown). There were no differences in human VEGF protein expression between control and PEITC-treated tumor samples as determined by Western blot analyses (Fig. 6B).

### PEITC affects cell attachment, but not proliferation in vitro

**Figure 8:**
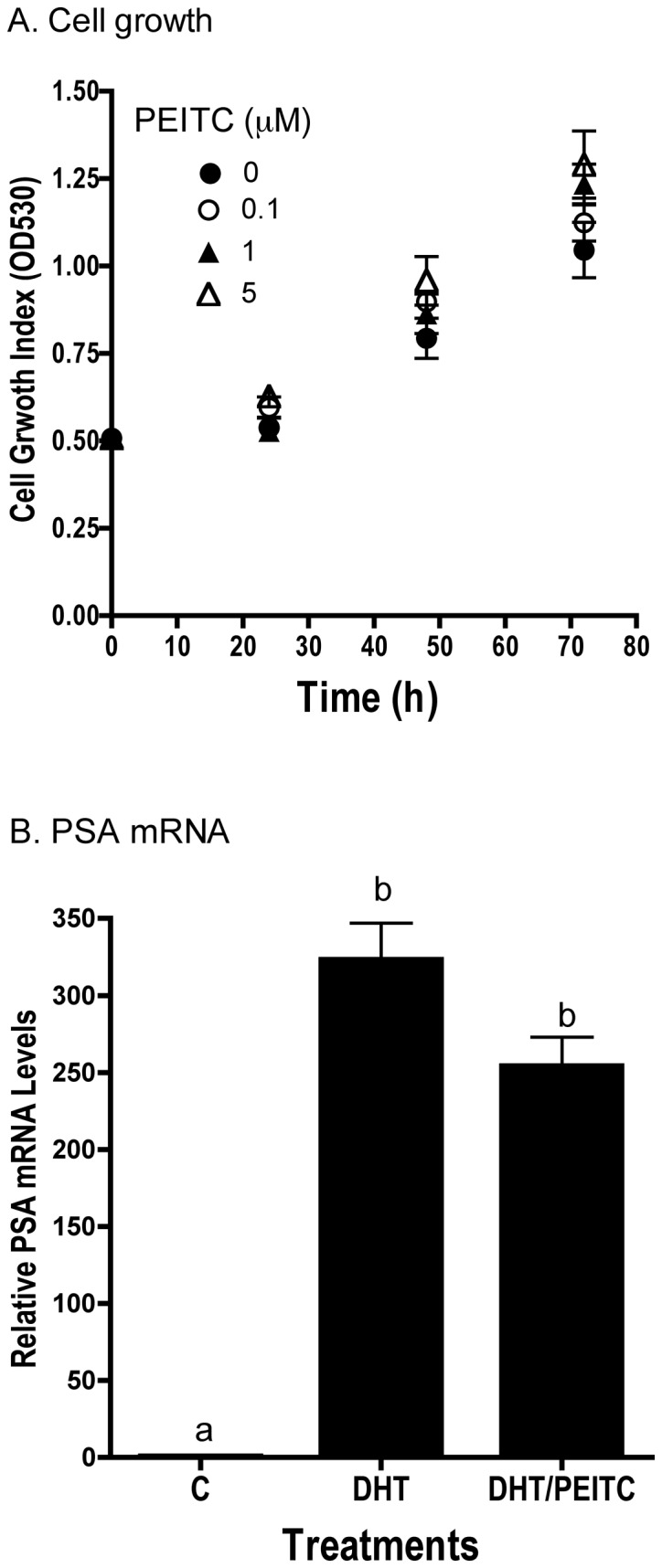
Effects of PEITC on LNCaP prostate cancer cell proliferation and responsiveness to DHT *in vitro*. (A) LNCaP cells were plated in 24-well plates; 24 h after plating the cells were treated with 0, 0.1, 1 or 5 μM PEITC for 75 h and cell growth determined as described in Materials and methods. Results are expressed as means ± SE (n=4). (B) Effects of PEITC on DHT-induction of PSA mRNA in cultured LNCaP cells. LNCaP cells were plated in 6-well plates, switched to media B 24 h later, and then 24 h later treated with control medium, 1 nM DHT, or 1 nM DHT + 5 μM PEITC for 48 h. RNA was harvested, and levels of PSA mRNA relative to G3PDH were quantified by real-time RT-PCR as described in Materials and methods. Results are expressed as means ± SE (n=3). Bars with different superscripts are significantly different from one another at p<0.05.

**Figure 9:**
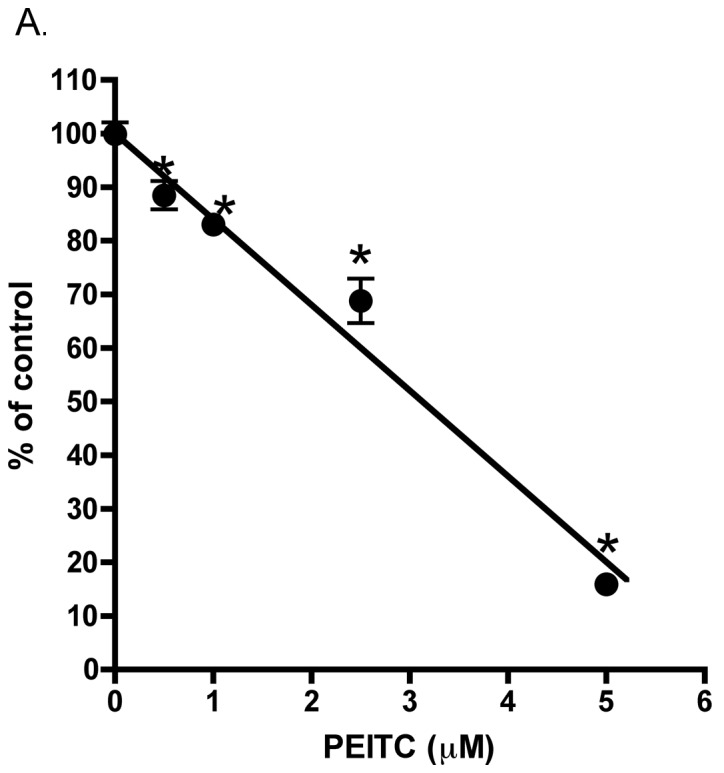

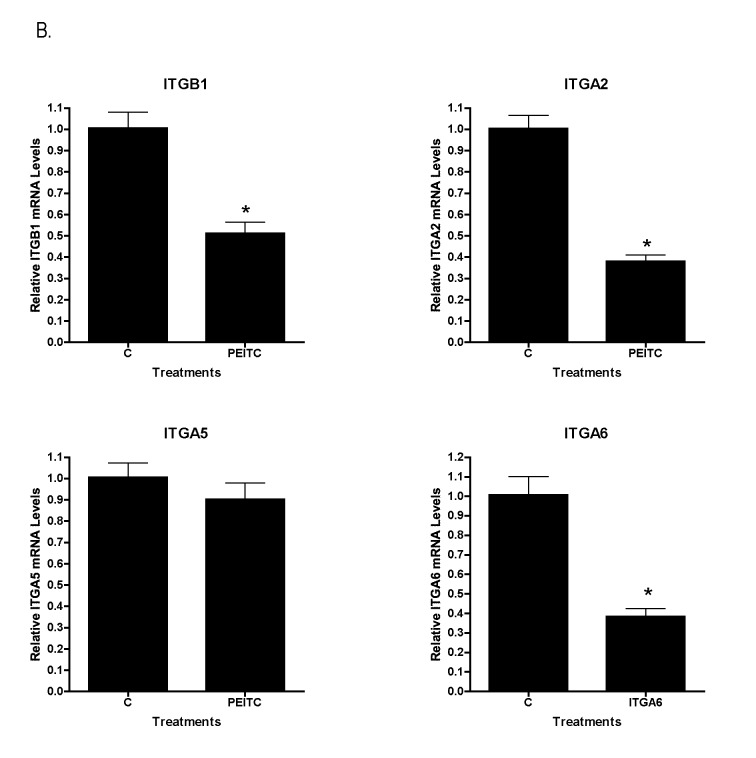
Effects of PEITC on LNCaP cell plating efficiency and intergrins expression *in vitro*. (A) Effects of PEITC on plating efficiency. LNCaP cells were plated in 24-well in presence of 0, 0.1, 1or 5 μM PEITC, after overnight incubation cell attached to the plate were determined using SRB method as described in Materials and methods. Results are expressed as the means ± SE (n=4). (B) Effects of PEITC on integrin expression. LNCaP cells were plated in 6-well plates, in presence or absence of 5 μM PEITC, after overnight incubation RNA from the attached cells were harvested, and levels of ITGB1, A2, A5, A6 mRNA relative to G3PDH were quantified by real-time RT-PCR as described in Materials and methods. Results are expressed as the means ± SE (n=3). Bars with asterisk are significantly different from vehicle control at p<0.05.

Given the small response of PEITC on angiogenesis we further asked what other pathways may account for inhibition of tumor growth using LNCaP cell culture model. Consistent with the lack of effects on cell proliferation and apoptosis *in vivo*, PEITC exerted minimal effects on LNCaP cells in culture. Concentrations of PEITC ≤5 μM had no effect on the growth of the LNCaP cells *in vitro* (Fig. 8A). In addition, cell cycle analysis showed no effect of PEITC on cell cycle progression or appearance of sub-G0 DNA, an indicator of apoptosis. Also consistent with *in vivo* results, PEITC did not affect DHT-induced increase in PSA mRNA levels *in vitro* (Fig. 8B). Interestingly, we found as illustrated in Fig. 9A, in the presence of PEITC there was a significant inhibition of LNCaP cell plating efficiency. This effect occurred at relatively low (0.5 μM) PEITC concentration. The integrin family proteins are known to play important roles in cell adhesion/attachment and prostate cancer development (23). We therefore asked whether alteration in integrins may correlate with a decreased in LNCaP cell plating efficiency. We found mRNA expression of integrins ITGB1, ITGA2 and ITGA6 was significantly inhibited in the PEITC (5 μM) treated LNCaP that attached to cell culture plate as compared to vehicle control (Fig. 9B). By contrast, ITGA5 mRNA expression was not affected by PEITC treatment (Fig. 9B).

## Discussion

The present study examined the anti-tumor effects of PEITC on androgen-responsive LNCaP human prostate cancer cells *in vitro* and *in vivo*. Three important pieces of information were revealed in this study: i) PEITC suppression of tumor growth is associated with diminished microvessel density *in vivo*. ii) PEITC at the dose and concentrations used in our study demonstrated no direct effects on LNCaP cancer cell growth *in vitro* and *in vivo*. iii) Cell attachment, but not proliferation was significantly affected by PEITC *in vitro*. The level of consumption of PEITC in the diet by nude mice was approximately 100–150 mg/kg body weight/day. At this level of PEITC consumption, a significantly lower tumor volume than in mice fed control diet was observed (Fig. 2). This growth inhibition was observed as early as 6 weeks after tumor cell injection (Fig. 2). The anti-tumor effect of PEITC on LNCaP cells is similar to observations by others in the androgen non-responsive PC-3 human prostate cancer cell line (14–16,24,25). However, the more interesting aspect of our study is the absence of direct effects of PEITC on cell proliferation and apoptosis in tumor *in vivo*. There were no differences in levels of PCNA, a proliferation marker, or DNA fragmentation (ApoTag), consistent with the lack of effects on LNCaP cell growth (Fig. 8A) and cell cycle (data not shown) we observed *in vitro*. Further supporting the immunohistochemical data, there were no significant differences in mRNA expression of PCNA (Fig. 5A) or another proliferation marker Ki-67 (Fig. 5B) and pro-apoptosis protein Bax (Fig. 5C), in tumor samples from control and PEITC-treated mice. The growth inhibitory and anti-apoptotic effects of PEITC *in vitro* reported by others in LNCaP cells (26,27) might arise from different culture conditions than ours. The differential effect of PEITC on apoptosis in PC-3 cell tumors and LNCaP cell tumors *in vivo* could be due to differences between an androgen non-responsive human prostate cancer model (PC-3 cells) and an androgen- responsive model (LNCaP cells). However, in addition to the cell proliferation end-point, PEITC also had no effect on PSA expression in LNCaP prostate tumors (Fig. 5B and C), corroborating the lack of PEITC effects on androgen-mediated pathways *in vitro* (Fig. 5A). These results support the notion that the inhibitory effect of PEITC on LNCaP cell tumor xenograft lay elsewhere.

The mechanisms underlying the inhibitory effects of PEITC on LNCaP cell-derived tumor growth may in part through altered angiogenesis (28,29). Microvessel density was significantly lower in tumors in mice consuming PEITC compared to control, as indicated by immunohistochemical staining for PECAM-1/CD31 (30), a marker for microvessel density (Fig. 7). However, this is different from a recent study that showed in PC-3 cells that PEITC at pharmacologically achievable concentrations inhibits angiogenesis *in vitro* and *ex vivo* by targeting VEGF (16). We did not detect changes in VEGF protein by immunohistochemistry, Western blot analysis or mRNA by real-time PCR (Fig. 5E and F). Alternatively, we propose that the effects of PEITC may occur right after tumor cell injection. In our experiment, animals were fed PEITC for two weeks before injection. This may create an environment where sufficient PEITC in circulation may already exist to prevent tumor cells to attach. Our cell culture results support this hypothesis. As illustrated in Fig. 9A, cell plating efficiency after overnight exposure to PEITC was significantly reduced in a concentration-dependent fashion that occurred at concentration as low as 0.5 μM. Furthermore, in those cells that attached to cell culture plate, we found a significantly lower mRNA expression of integrin family proteins ITGB1, ITGA2, ITGA6 in cells exposed to PEITC (Fig. 9B). ITGB1 is a common subunit for fibronectin, laminin and collagen receptors (31–33). ITGA2 is a subunit for collagen receptor (32) and ITGA6 is a subunit for laminin receptor (33). These integrins are known to play an important role in cell adhesion/attachment and prostate cancer development (23,31–33). Therefore, it is possible that this inhibition of cell adhesion/attachment-related protein may decrease initial number of cells established at the injection site and lead to smaller tumor size in PEITC-fed animals. Thus, the tumor growth inhibitory effects of PEITC in our system may be indirect. The effect of PEITC on integrins appeared to be specific as ITGA5 mRNA levels did not alter (Fig. 9B). Additional studies are warranted to further support this hypothesis.

Cruciferous vegetables are unique among the foods consumed in the diet in that they have been consistently shown to have beneficial effects in the prevention of various forms of cancers, including prostate (10,34–37). These effects may be due to modulation of multiple pathways by various ITCs present in these vegetables. In addition to the inhibition of angiogenesis by PEITC observed in this study, indoles, another family of phytochemicals found in cruciferous vegetables, are known to modulate androgen-responsive pathways (38). Moreover, sulforaphane, also found in cruciferous vegetables, may offer protection against carcinogens through activation of xenobiotic metabolism pathways (39). It would be of interest to examine combinations of compounds, as well as temporal effects during carcinogenesis, to further elucidate the benefits of cruciferous vegetables.

In conclusion, the effects of PEITC on androgen-responsive LNCaP human prostate cancer cells in the present study appeared to differ from those seen in androgen-non-responsive PC-3 human prostate cancer cells (16,25). The mechanisms underlying the inhibitory effects of PEITC on growth of LNCaP xenografts appeared to be independent of cell proliferation or apoptosis and may be through modulation of the tumor microenvironment by regulation of attachment and/or angiogenesis.

## References

[1] Ozasa K, Nakao M, Watanabe Y, Hayashi K (2004). Serum phyto-estrogens and prostate cancer risk in a nested case-control study among Japanese men. Cancer Sci.

[2] Boyle P, Levi F, Lucchini F, La Vecchia C (1993). Trends in diet-related cancers in Japan: a conundrum?. Lancet.

[3] Morton MS, Griffiths K, Blacklock N (1996). The preventive role of diet in prostatic disease. Br J Urol.

[4] Cook LS, Goldoft M, Schwartz SM, Weiss NS (1999). Incidence of adenocarcinoma of the prostate in Asian immigrants to the United States and their descendants. J Urol.

[5] Stellman SD, Wang QS (1994). Cancer mortality in Chinese immigrants to New York City. Comparison with Chinese in Tianjin and with United States-born whites. Cancer.

[6] Schuman LM, Mandel JS (1980). Epidemiology of prostatic cancer in blacks. Prev Med.

[7] Whittemore AS, Lele C, Friedman GD, Stamey T (1995). Prostate-specific antigen as predictor of prostate cancer in black men and white men. J Natl Cancer Inst.

[8] Singh RP, Agarwal R (2003). Tumor angiogenesis: a potential target in cancer control by phytochemicals. Curr Cancer Drug Targets.

[9] Zarkovic N, Vukovic T, Loncaric I, Miletic M (2001). An overview on anticancer activities of the Viscum album extract Isorel. Cancer Biother Radiopharm.

[10] Hecht SS (1995). Chemoprevention by isothiocyanates. J Cell Biochem.

[11] Zhang Y, Kensler TW, Cho CG, Posner GH, Talalay P (1994). Anticarcinogenic activities of sulforaphane and structurally related synthetic norbornyl isothiocyanates. Proc Natl Acad Sci USA.

[12] Yu R, Mandlekar S, Harvey KJ, Ucker DS, Kong AN (1998). Chemo-preventive isothiocyanates induce apoptosis and caspase-3-like protease activity. Cancer Res.

[13] Xu K, Thornalley PJ (2001). Involvement of glutathione metabolism in the cytotoxicity of the phenethyl isothiocyanate and its cysteine conjugate to human leukaemia cells *in vitro*. Biochem Pharmacol.

[14] Xiao D, Singh SV (2002). Phenethyl isothiocyanate-induced apoptosis in p53-deficient PC-3 human prostate cancer cell line is mediated by extracellular signal-regulated kinases. Cancer Res.

[15] Xu C, Shen G, Chen C, Gelinas C, Kong AN (2005). Suppression of NF-kappaB and NF-kappaB-regulated gene expression by sulforaphane and PEITC through IkappaBalpha, IKK pathway in human prostate cancer PC-3 cells. Oncogene.

[16] Xiao D, Singh SV (2007). Phenethyl isothiocyanate inhibits angiogenesis *in vitro* and *ex vivo*. Cancer Res.

[17] Morse MA, Reinhardt JC, Amin SG, Hecht SS (1990). Effect of dietary aromatic isothiocyanates fed subsequent to the administration of 4-(methylnitrosamino)-1-(3-pyridyl)-1-butanone on lung tumorigenicity in mice. Cancer Lett.

[18] Zhou JR, Gugger ET, Tanaka T, Guo Y (1999). Soybean phytochemicals inhibit the growth of transplantable human prostate carcinoma and tumor angiogenesis in mice. J Nutr.

[19] Wang TT, Hudson TS, Wang TC, Remsberg CM (2008). Differential effects of resveratrol on androgen-responsive LNCaP human prostate cancer cells *in vitro* and *in vivo*. Carcinogenesis.

[20] Hunter AL, Choy JC, Granville DJ (2005). Detection of apoptosis in cardiovascular diseases. Methods Mol Med.

[21] Cher ML, Chew K, Rosenau W, Carroll PR (1995). Cellular proliferation in prostatic adenocarcinoma as assessed by bromodeoxyuridine uptake and Ki-67 and PCNA expression. Prostate.

[22] Takahashi Y, Lavigne JA, Hursting SD, Chandramouli GV (2006). Molecular signatures of soy-derived phytochemicals in androgen-responsive prostate cancer cells: a comparison study using DNA microarray. Mol Carcinog.

[23] Goel HL, Alam N, Johnson IN, Languino LR (2009). Integrin signaling aberrations in prostate cancer. Am J Transl Res.

[24] Kim JH, Xu C, Keum YS, Reddy B (2006). Inhibition of EGFR signaling in human prostate cancer PC-3 cells by combination treatment with beta-phenylethyl isothiocyanate and curcumin. Carcinogenesis.

[25] Xiao D, Johnson CS, Trump DL, Singh SV (2004). Proteasome-mediated degradation of cell division cycle 25C and cyclin-dependent kinase 1 in phenethyl isothiocyanate-induced G2-M-phase cell cycle arrest in PC-3 human prostate cancer cells. Mol Cancer Ther.

[26] Chen YR, Han J, Kori R, Kong AN, Tan TH (2002). Phenylethyl isothiocyanate induces apoptotic signaling via suppressing phosphatase activity against c-Jun N-terminal kinase. J Biol Chem.

[27] Xiao D, Herman-Antosiewicz A, Antosiewicz J, Xiao H (2005). Diallyl trisulfide-induced G(2)-M phase cell cycle arrest in human prostate cancer cells is caused by reactive oxygen species-dependent destruction and hyperphosphorylation of Cdc 25 C. Oncogene.

[28] Van Moorselaar RJ, Voest EE (2002). Angiogenesis in prostate cancer: its role in disease progression and possible therapeutic approaches. Mol Cell Endocrinol.

[29] Weidner N, Carroll PR, Flax J, Blumenfeld W, Folkman J (1993). Tumor angiogenesis correlates with metastasis in invasive prostate carcinoma. Am J Pathol.

[30] Cao G, O'Brien CD, Zhou Z, Sanders SM (2002). Involvement of human PECAM-1 in angiogenesis and *in vitro* endothelial cell migration. Am J Physiol Cell Physiol.

[31] Zeng ZZ, Jia Y, Hahn NJ, Markwart SM, Rockwood KF, Livant DL (2006). Role of focal adhesion kinase and phosphatidylinositol 3′-kinase in integrin fibronectin receptor-mediated, matrix metalloproteinase-1-dependent invasion by metastatic prostate cancer cells. Cancer Res.

[32] Hall CL, Dubyk CW, Riesenberger TA, Shein D, Keller ET, van Golen KL (2008). Type I collagen receptor (alpha2beta1) signaling promotes prostate cancer invasion through RhoC GTPase. Neoplasia.

[33] Sroka IC, Anderson TA, McDaniel KM, Nagle RB, Gretzer MB, Cress AE (2010). The laminin binding integrin alpha6beta1 in prostate cancer perineural invasion. J Cell Physiol.

[34] Morse MA, Zu H, Galati AJ, Schmidt CJ, Stoner GD (1993). Dose-related inhibition by dietary phenethyl isothiocyanate of esophageal tumorigenesis and DNA methylation induced by N-nitrosomethylbenzylamine in rats. Cancer Lett.

[35] Stoner GD, Morrissey DT, Heur YH, Daniel EM (1991). Inhibitory effects of phenethyl isothiocyanate on N-nitrosobenzylmethylamine carcinogenesis in the rat esophagus. Cancer Res.

[36] Wattenberg LW (1981). Inhibition of carcinogen-induced neoplasia by sodium cyanate, tert-butyl isocyanate, and benzyl isothiocyanate administered subsequent to carcinogen exposure. Cancer Res.

[37] Wattenberg LW (1987). Inhibitory effects of benzyl isothiocyanate administered shortly before diethylnitrosamine or benzo[a]pyrene on pulmonary and forestomach neoplasia in A/J mice. Carcinogenesis.

[38] Hsu JC, Zhang J, Dev A, Wing A (2005). Indole-3-carbinol inhibition of androgen receptor expression and downregulation of androgen responsiveness in human prostate cancer cells. Carcinogenesis.

[39] Talalay P, Fahey JW (2001). Phytochemicals from cruciferous plants protect against cancer by modulating carcinogen metabolism. J Nutr.

